# Study on the Selective Behavior of *Brachymystax tsinlingensis* Li, 1966 (Order: Saloniformes, Family: Salmonidae) on Substrate Color and Type

**DOI:** 10.3390/ani15142089

**Published:** 2025-07-15

**Authors:** Lin Zhang, Rongqun Song, Jian Shao

**Affiliations:** 1 Laboratory of Fishery Resources and Environmental Protection, College of Animal Science, Guizhou University, Guiyang 550025, China; zhanglin012656@163.com (L.Z.); songrongqun2021@163.com (R.S.); 2 The Key Laboratory of Animal Genetics, Breeding and Reproduction in the Plateau Mountainous Region, Ministry of Education, College of Animal Science, Guizhou University, Guiyang 550025, China; 3 Special Fisheries Research Institute, Guizhou University, Guiyang 550025, China

**Keywords:** *Brachymystax tsinlingensis*, substrate, color, type, behavior, selectivity

## Abstract

Substrate is an important component of a fish’s habitat environment. Fish preferences for substrate influence their growth and development, feeding, hiding, schooling, and reproduction. To explore the habitat preference of *Brachymystax tsinlingensis*, this study was conducted on the preferences of B. pre-smolts, post-smolts, and juveniles for three substrate colors, white, blue, and black, and four substrate types, sand (<0.5 cm in diameter), small gravel (1–2 cm in diameter), medium gravel (5–7 cm in diameter), and large gravel (12–16 cm in diameter), which were investigated in light (10–60 lx) and dark (no light) environments. The results showed that the individual populations of *B. tsinlingensis* in three periods of time had a clear preference for substrate color, preferring black substrate and staying away from blue substrate under both light and dark environmental conditions; *B. tsinlingensis* pre-smolts and post-smolts preferred sandy substrate, and juveniles preferred medium gravel substrate. The choice of substrate color and substrate type by *B. tsinlingensis* is a manifestation of the living environment characteristics of this species, which is conducive to their hiding, better avoidance of enemies, and improvement of their survival rate. Based on the research results and the early biological characteristics of *B. tsinlingensis*, it is recommended to use black or dark substrate during the cultivation of *B. tsinlingensis* fry. During the larval fish stage, a sandy substrate environment should be provided. During the juvenile fish stage, a medium-gravel environment should be provided.

## Simple Summary

*Brachymystax tsinlingensis* is a unique cold-water fish locally distributed in China. As early as 1988, it has been included in the national second-class protection of wildlife; its protection is crucial, but because of its sensitive habitat and unique habits—especially on the choice of substrate environment is extremely harsh—the substrate environment was determined to play an important role in the protection of *B. tsinlingensis*. For this reason, this study used the flume experiment method to observe the substrate selection behavior of *B. tsinlingensis*, aiming to clarify the characteristics of substrate selection behavior of *B. tsinlingensis*, grasp its habitat preferences, and provide technical guidance for environmental selection in its seedling cultivation and resource restoration process.

## 1. Introduction

The selective behavior of different vertebrates is increasingly of interest to researchers [1,2]. Different species of animals recognize their home range by a number of criteria, which ensures their sustainable existence in the environment. Identification of these criteria ensures sustainable management of animal behavior in natural and artificial habitats. Fish, as the most important aquatic organisms, have habitat substrate characteristics in different water bodies based on their complex composition of non-living and living organisms, of which mud, sand, and gravel are non-living, and water plants, seaweeds, etc., are living organisms [3,4]. Differences in substrate can have a significant impact on the growth, reproduction, aggregation, predation, and other life activities of fish [5,6,7,8,9,10]. In the complex environment of habitat substrate, fish will make adaptive choices according to the functions provided by different substrate and their own preferences. For example, juvenile *Acipenser sinensis* (Gray, 1835) choose muddy and sandy substrate for smooth overwintering [11]. The dynamic game between predators and prey often manifests as a coevolution of camouflage and anti-camouflage. Prey typically reduce the probability of being eaten through protective coloration (matching the environment), morphology, or behavior (hiding behavior), while predators use contrasting colors to more easily identify food. This “deceptive adaptation” shapes differences in habitat selection [12,13,14]. *Anguilla japonica* (Temminck & Schlegel, 1846) in the Yangtze River estuary choose black substrate for hiding in order to avoid enemies and predation [8], *Acipenser schrenckii* (Brandt, 1869) choose a white substrate in order to facilitate the discovery and predation of small bait [15]; light intensity represents the brightness of the habitat. Areas with strong light intensity are mostly shallow water areas where sunlight penetrates fully, while areas with weak light intensity are mostly deep-water areas where light intensity is significantly reduced. Under strong light induction, Plectognath fish will select different habitats [16]. *Glyptosternum maculatum* (Regan, 1905) choose different substrate colors under different light conditions, choosing black substrate under low-light compensation (10 ± 2 lx) conditions and refraining from making a choice under strong-light compensation (400 ± 50 lx) conditions [17]; these behaviors favor their improved survival. The same kind of substrate provides very different roles for different fishes, and even the roles provided by the substrate are different in different developmental periods of the same fish [3]; the substrate has also become one of the key constraints for artificial breeding and resource augmentation. Therefore, the study of fish habitat environment is of great significance in practice, particularly for the cultivation and resource enhancement of rare fish species. The provision of a suitable substrate environment is conducive to the large-scale production of fry and the improvement of survival rate.

*Brachymystax tsinlingensis* Li 1966 belongs to Salmoniformes, Salmonidae, *Brachymystax*, alias Flowerfish (Shaanxi), Plum Fish (Gansu), etc. It is a cold-water fish endemic to China. They are distributed in the Black River, Stone River, Chishui River, and Taibai River sections of the Taibai Peak of the Qinling Mountains, as well as in the tributaries of the Qian River in Longxian County and the tributaries of the Wei River in Gansu [18]. Due to the strong stress, harsh habitat conditions, and slow growth [19], coupled with the effects of anthropogenic overfishing and other impacts, which led to a sharp decline in the wild populations of *B. tsinlingensis*, the trend of individual miniaturization is obvious, and the destruction of the germplasm resources is serious; it has been listed as a national second-class protected animal [20]. In recent years, some scholars have successively conducted comprehensive research on the early development [21], age, growth [22], genetics, and reproduction [23] of *B. tsinlingensis*. Preliminary breakthroughs have been made in the artificial propagation technology of *B. tsinlingensis*, but the survival rate of fry cultivation is low; at the same time, we have found that substrate condition affects one of the key factors of fry survival in the process of broodstock rearing, but the studies on the correlation of the substrate type of *B. tsinlingensis* have been very scarce so far. Therefore, in this study, we used transparent aquariums to observe the selection behavior of *B. tsinlingensis* on substrate color and substrate type, with the aim of understanding the habitat preferences of *B. tsinlingensis*, improving the breeding, and increasing the survival rate of *B. tsinlingensis* fry.

## 2. Materials and Methods

### 2.1. Laboratory Animals

The experiments were conducted from May to September 2023 at the *B. tsinlingensis* artificial breeding experimental base in the Qinling Mountain region (Taibai County, Baoji City, Shaanxi Province, China). Fish used in the experiment were obtained from catalytic incubation of Chinook salmon fry, and then healthy pre-smolts, post-smolts, and juveniles were randomly selected from the same batch. The standard length and weight of the experimental fish were measured (Table 1). The experimental fish were transferred to the experimental tank to acclimatize for 10 days prior to the experiment to avoid environmental stresses as a way to reduce the experimental error. Real-time monitoring of the experimental fish feeding during the acclimatization period revealed that the feeding behavior of the experimental fish was obvious on the 5th day, and the feeding effect was optimal, which could be recognized as having fully adapted to the experimental environment. The experimental fish were not fed 2 h before the start of the experiment to avoid feeding factors affecting the experimental results, the aquarium was cleaned and sterilized, and then the experimental water was added (water depth 20.06–21.50 cm). The water quality parameters of the experimental water were measured by a YSI ProPlus (Yellow Springs, OH, USA) multi-parameter water quality meter, the water temperature was (13.65 ± 1.88) °C, dissolved oxygen was (7.56 ± 0.83) mg/L, and the pH was 7.08 ± 0.16.

### 2.2. Experimental Setup

#### 2.2.1. Experimental Setup for Substrate Color

**Figure 1:**
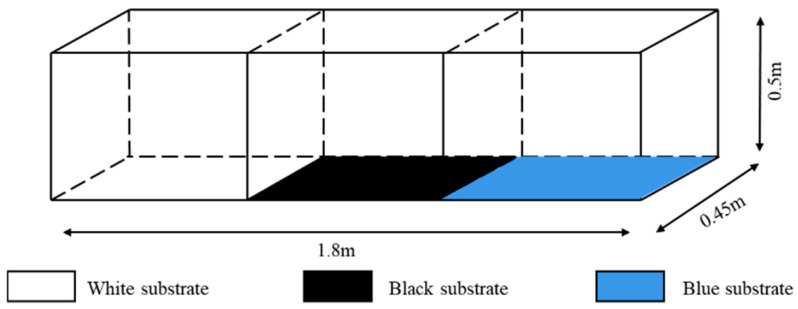
Sketch map for substrate color preference test (dark situation).

**Figure 2:**
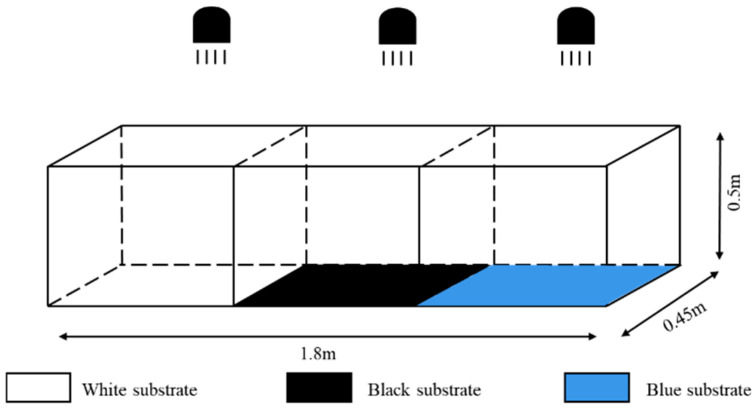
Sketch map for substrate color preference test (illuminated situation).

The same area of white, black, and blue opaque-color cloth (0.60 m long × 0.45 m wide) was laid on the bottom of the aquarium, the aquarium was divided into three areas of equal size, and then the experiment was conducted in the light and dark environments, respectively. The dark environment device was conducted under no light conditions (Figure 1), and the light environment device used three 15 W incandescent lamps as the only light source placed directly above the aquarium; the position between the lamps was adjusted so that the light intensity of the three equal-area areas fell within the optimal light range (10~60 lx) (Figure 2). Substrate positions were exchanged at the end of each set of experiments to eliminate positional adaptation errors [24].

#### 2.2.2. Substrate Type Experimental Setup

**Figure 3:**
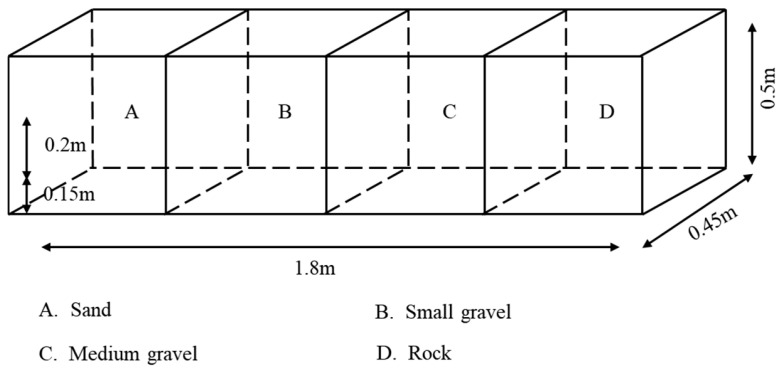
Sketch map for substrate type preference test (dark situation).

**Figure 4:**
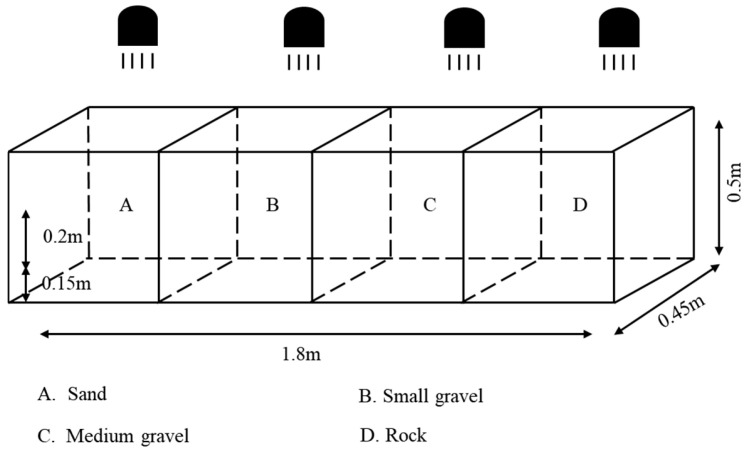
Sketch map for substrate type preference test (illuminated situation).

The bottom of the aquarium is divided into 4 areas of equal size, each with an equal substrate depth (20–21 cm), using large gravel (12–16 cm in diameter, with gaps filled with small gravel), medium gravel (5–7 cm in diameter), small gravel (1–2 cm in diameter), and sand (<0.05 cm in diameter) laid on the bottom. The dark environment device was conducted under no light conditions (Figure 3), and the light environment device used four 15 W incandescent lamps as the only light source placed directly above the aquarium; the position between the lamps was adjusted so that the light intensity of the four equal-area areas fell within the optimal light range (10~60 lx) (Figure 4). Substrate positions were exchanged at the end of each set of experiments to eliminate positional adaptation errors [24].

### 2.3. Experimental Methods

Individual and population experiments on substrate color and individual and population experiments on substrate type were conducted under light and dark environmental conditions for pre-smolts, post-smolts, and juveniles sequentially, respectively. 1. Individual experiment: Randomly selected 16 experimental fish, put 1 fish into the aquarium each time, put 1 fish into the center of the aquarium each time, and after 2 min of acclimatization, used a video camera (acA1920, 155uc NIR, Balser, Ahrensburg, Germany) to record for 5 min (30 frames/s) and then fished out the experimental fish. Then the next experiment was conducted, which was carried out in the same way. The time of stay of each fish in each area was counted, and the average of the percentage of time of stay of the experimental fish in each area was used as an indicator of individual selectivity for substrate color and substrate type. 2. Population experiment: A total of 240 experimental fish were randomly selected; 15 fish were put into each group at the same time for the experiment; after acclimatization for 2 min, the number of experimental fish in different areas was recorded once every 30 s interval, 10 times for each group; and finally all the experimental fish were fished out and the next group of experiments was carried out in the same way. The percentage of the number of experimental fish in each area at each recording was calculated, and the average of the percentage of the number in each area was used as an indicator of the selectivity of the population to different substrate colors and substrate types [24].

### 2.4. Data Processing

The experiment is standardized by the head of the experimental fish entering a certain area, which indicates that the experimental fish is in that area, and timing or counting can be started. One-way ANOVA and nonparametric tests were performed using SPSS 25.0 software; the experimental results are expressed as mean ± standard deviation (mean ± SD). Duncan’s multiple comparison test and the Kruskal–Wallis test were used to analyze the significance of differences between groups.

## 3. Results

### 3.1. Substrate Color Selection Experiment

#### 3.1.1. Results of Individual Experiments in Light Environment

**Figure 5:**
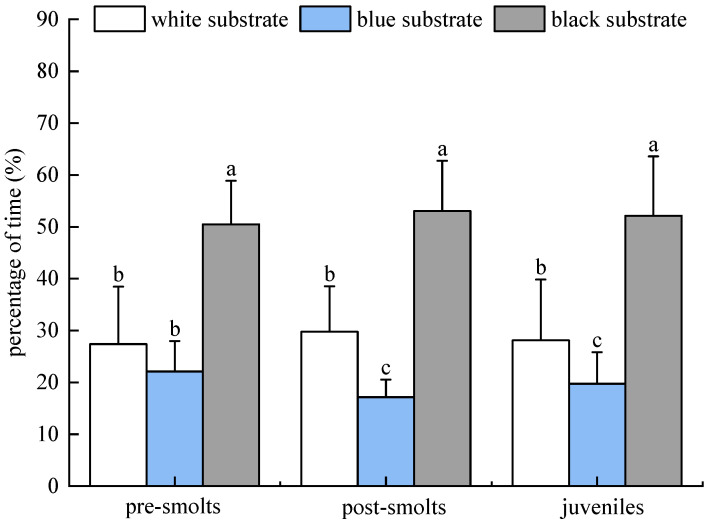
*B. tsinlingensis* substrate color (individual experiment in light environment). (N = 16, different letters mean significant difference *p* < 0.05).

The percentage of time that *B. tsinlingensis* pre-smolts, post-smolts, and juveniles spent in areas of three substrate colors (white, blue, and black) in individual experiments in light environments (10–60 lx) is as follows: The percentage of dwell time of pre-smolts in the area of three substrate colors (white, blue, and black) occurred in the following order: (27.40 ± 11.07)%, (22.11 ± 5.86)%, (50.49 ± 8.39)%. The percentage of dwell time of the post-larvae occurred in the following order: (29.78 ± 8.77)%, (17.16 ± 3.38)%, (53.07 ± 9.66)%. The percentage residence time of juvenile fish in three substrate color areas occurred in the following order: (28.11 ± 11.74)%, (19.76 ± 6.05)%, (52.13 ± 11.43)%. Pre-smolts spent the longest time in the black substrate area (*p* < 0.05) and the shortest time in the blue substrate area; post-smolts and juveniles spent the longest time in the black substrate area, with the percentage of time spent in the black substrate area significantly higher than that of the white substrate area (*p* < 0.05), and the percentage of time spent in the white substrate area significantly higher than that of the blue substrate area (*p* < 0.05). Therefore, *B. tsinlingensis* had a clear substrate preference, preferring black substrate and fleeing blue substrate (Figure 5).

#### 3.1.2. Results of Individual Experiments in Dark Environment

**Figure 6:**
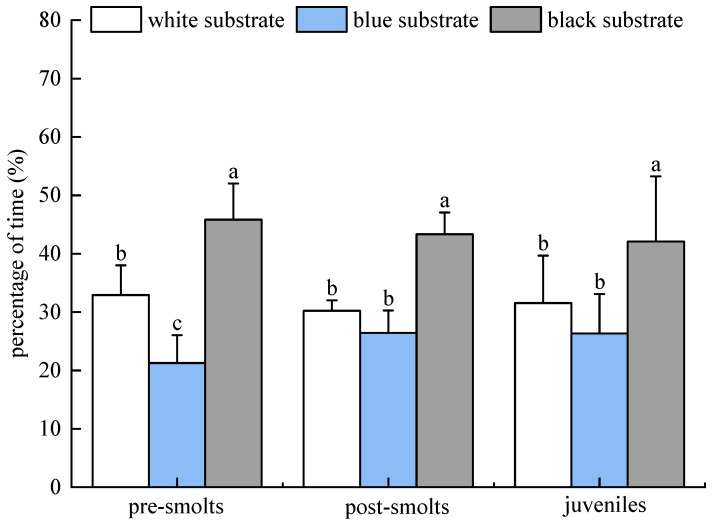
*B tsinlingensis* substrate color (individual experiment in dark environment). (N = 16, different letters mean significant difference *p* < 0.05).

The percentage of time that *B. tsinlingensis* pre-smolts, post-smolts, and smolts spent in areas of three substrate colors (white, blue, and black) in individual experiments in dark is as follows. The percentage of dwell time of pre-smolts in the area of three substrate colors (white, blue, and black) occurred in the following order: (32.91 ± 5.08)%, (21.27 ± 4.79)%, (45.82 ± 6.19)%. The percentage of dwell time in the post-larvae occurred in the following order: (30.22 ± 1.80)%, (26.44 ± 3.84)%, (43.33 ± 3.74)%. The percentage residence time of juvenile fish in three substrate color areas occurred in the following order: (31.56 ± 8.10)%, (26.36 ± 6.73)%, (42.09 ± 11.17)%. *B. tsinlingensis* pre-smolts, post-smolts, and juveniles spent significantly more time in the black substrate than in the other areas (*p* < 0.05), followed by the white area and the blue area (*p* < 0.05). Therefore, *B. tsinlingensis* had a clear substrate preference, preferring the black substrate and fleeing the blue substrate (Figure 6).

In both light and dark environments, individuals of *B. tsinlingensis* showed the same and obvious preference for substrate color, preferring black substrate and escaping from blue substrate.

#### 3.1.3. Results of Population Experiments in Light Environment

**Figure 7:**
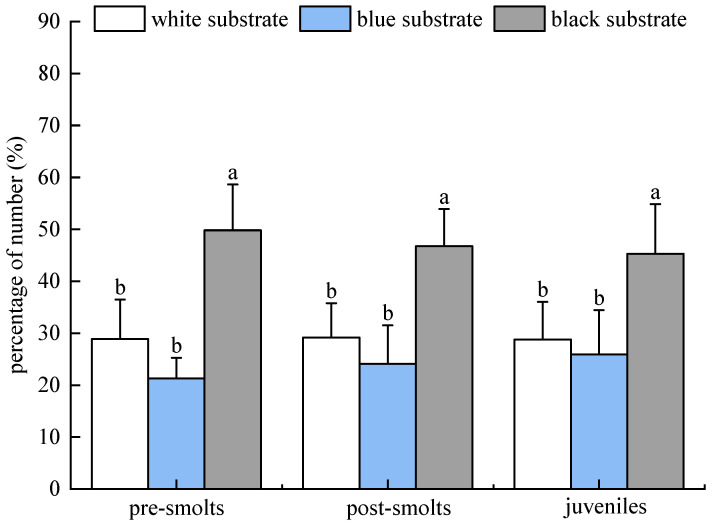
*B*. *tsinlingensis* substrate color (population experiment in light environment). (N = 160, different letters mean significant difference *p* < 0.05).

The percentages of the number of tails in the distribution of the population of *B tsinlingensis* pre-smolts, post-smolts, and juveniles in the three substrate color (white, blue, and black) areas in the light environment (10–60 lx) are as follows: The percentage of the number of tails distributed in the three substrate color areas of white, blue, and black for pre-smolts occurred in the following order: (28.89 ± 7.59)%, (21.29 ± 3.96)%, (49.82 ± 8.82)%. The percentage of the number of tails distributed in the post-smolts occurred in the following order: (29.16 ± 6.62)%, (24.09 ± 7.43)%, (46.76 ± 7.17)%. The percentage of the number of tails distributed in the three substrate color areas for juvenile fish occurred in the following order: (28.80 ± 7.23)%, (25.91 ± 8.53)%, (45.29 ± 9.56)%. *B. tsinlingensis* in all three periods had the highest (*p* < 0.05) number of distributed tails in the black substrate, the second highest number of distributed tails in the white region, and the lowest number of distributed tails in the blue region. Therefore, *B. tsinlingensis* had a clear preference for substrate, preferring black substrate and staying away from blue substrate (Figure 7).

#### 3.1.4. Results of Population Experiments in Dark Environment

**Figure 8:**
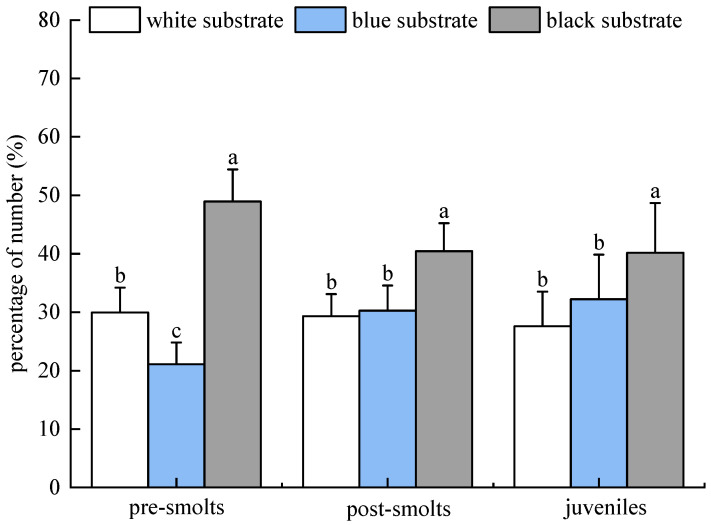
*B. tsinlingensis* substrate color (population experiment in dark environment). (N = 160, different letters mean significant difference *p* < 0.05).

The percentages of the number of tails in the distribution of the population of *B. tsinlingensis* pre-smolts, post-smolts, and juveniles in the three substrate colors (white, blue, and black) in the dark are as follows: The percentage of number of tails distributed in the three substrate color areas of white, blue, and black for pre-smolts occurred in the following order: (29.96 ± 4.25)%, (21.11 ± 3.69)%, (48.93 ± 5.50)%. The percentage number of tails distributed in the post-smolts occurred in the following order: (29.29 ± 3.80)%, (30.27 ± 4.30)%, (40.44 ± 4.80)%. The percentage of number of tails distributed in the three substrate color areas for juvenile fish occurred in the following order: (27.60 ± 5.92)%, (32.22 ± 7.63)%, (40.18 ± 8.51)%. The number of tails distributed in the black substrate area was significantly higher (*p* < 0.05) and the number of tails distributed in the white area was significantly higher (*p* < 0.05) than that in the blue substrate for the pre-smolts population; the number of tails distributed in the black substrate area was the highest (*p* < 0.05) and the number of tails distributed in the white and blue areas was not significant (*p* > 0.05) for the post-smolt population and juvenile population. Therefore, *B*. *tsinlingensis* had a clear substrate preference, preferring black substrate, while pre-smolts escaping from blue substrate (Figure 8).

In both light and dark environments, *B*. *tsinlingensis* populations showed similar substrate color preferences, preferring black substrate and fleeing blue substrate.

### 3.2. Substrate Type Selection Experiment

#### 3.2.1. Results of Individual Experiments in Light Environment

**Figure 9:**
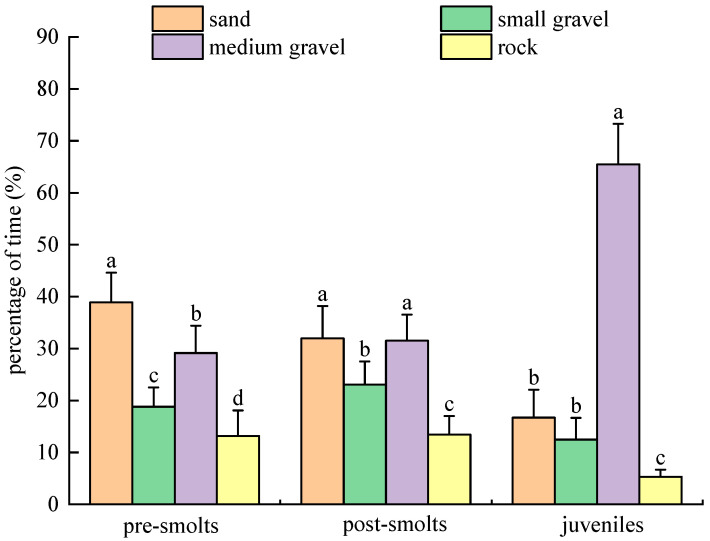
*B. tsinlingensis* substrate types (individual experiment in light environment). (N = 16, different letters mean significant difference *p* < 0.05).

In individual experiments with substrate types in the light environment (10–60 lx) for *B. tsinlingensis*, the percent of residence time that pre-smolts, post-smolts, and juveniles spent in the area of four substrate types (rock: 12–16 cm diameter; medium gravel: 5–7 cm diameter; small gravel: 1–2 cm diameter; and sand: <0.5 cm diameter) was as follows: pre-smolts of residence time by prelitters in each substrate area were (13.17 ± 4.93)% for rock, (29.15 ± 5.25)% for medium gravel, (18.79 ± 3.72)% for small gravel, and (38.90 ± 5.72)% for sand; post-smolts of residence time by prelitters in each substrate area were (13.44 ± 3.56)% for rock, (31.52 ± 5.02)% for medium gravel, (23.08 ± 4.45)% for small gravel, and (31.96 ± 6.24)% for sand; the percentage of time spent by juvenile fish in the four substrate type areas was (5.33 ± 1.36)% for rock, (65.48 ± 7.81)% for medium gravel, (12.48 ± 4.17)% for small gravel, and (16.71 ± 5.36)% for sand. The percentage of time spent by pre-smolts in the sandy substrate area was significantly (*p* < 0.05) higher than in the other areas; the percentage of time spent in areas of rock substrate was significantly lower (*p* < 0.05) than in other areas. The percentage of time spent by post-smolts in areas of medium gravel and sandy substrate was significantly higher (*p* < 0.05) than in small gravel, and not significant (*p* > 0.05) between medium gravel and sandy substrate; the least amount of time was spent in the rock substrate. Juveniles spent significantly more time in the medium gravel area (*p* < 0.05) and significantly less time in the rock area (*p* < 0.05). As *B. tsinlingensis* grew and developed, juveniles spent dramatically more time in the medium gravel substrate area than pre-smolts and post-smolts (*p* < 0.05); they spent significantly less time on the sandy bottom than pre-smolts and post-smolts (*p* < 0.05). Therefore, under light conditions, pre-smolts preferred sandy substrate; post-smolts preferred sandy and medium gravel substrate; juveniles preferred medium gravel substrate; and *B. tsinlingensis* of all three periods tended to avoid large gravel substrate (Figure 9).

#### 3.2.2. Results of Individual Experiments in Dark Environment

**Figure 10:**
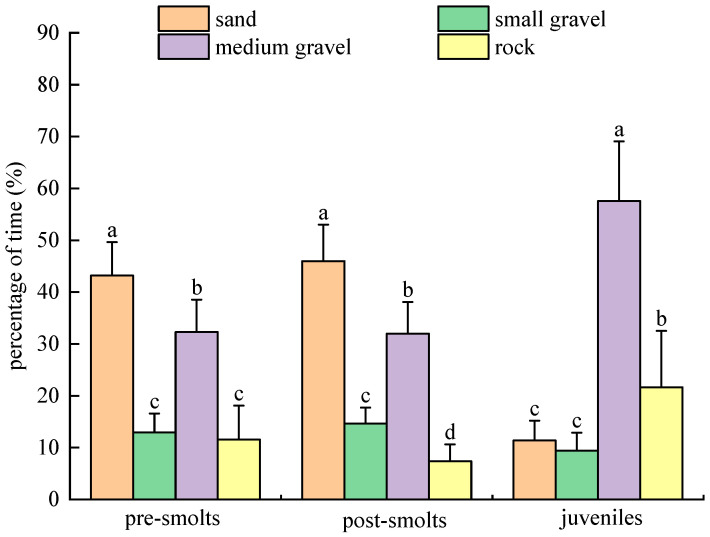
*B. tsinlingensis* substrate types (individual experiment in dark environment). (N = 16, different letters mean significant difference *p* < 0.05).

In individual experiments with substrate types in the dark for *B. tsinlingensis*, the percent of residence time that pre-smolts, post-smolts, and juveniles spent in the area of four substrate types (rock: 12–16 cm diameter; medium gravel: 5–7 cm diameter; small gravel: 1–2 cm diameter; and sand: <0.5 cm diameter) was as follows: pre-smolts of residence time by prelitters in each substrate area were (11.56 ± 6.52)% for rock, (32.31 ± 6.25)% for medium gravel, (12.94 ± 3.64)% for small gravel, and (43.19 ± 6.43)% for sand; post-smolts of residence time by prelitters in each substrate area was (7.40 ± 3.24)% for rock, (31.98 ± 6.10)% for medium gravel, (14.63 ± 3.12)% for small gravel, and (46.00 ± 7.04)% for sand; the percentage of time spent by juvenile fish in the four substrate type areas was (21.65 ± 10.86)% for rock, (57.56 ± 11.49)% for medium gravel, (9.40 ± 3.49)% for small gravel, and (11.40 ± 3.82)% for sand. Pre-smolts spent the most significant excess time in sandy substrate areas (*p* < 0.05) over medium and small gravel, and the least time in rock areas. Post-smolts spent significantly more time in the sandy substrate area (*p* < 0.05) and significantly less time in the rock area (*p* < 0.05) than in the other areas. Juveniles spent a significantly higher (*p* < 0.05) percentage of time in the medium gravel substrate area than in the other areas, and there was no significant difference (*p* > 0.05) between the sandy substrate and the small gravel substrate. With growth and development, juveniles differed significantly (*p* < 0.05) from smolts (pre-smolts and post-smolts), with juveniles spending a rapidly increasing amount of time in the medium- and large-gravel substrate areas, significantly more than pre-smolts and post-smolts (*p* < 0.05), and significantly less time in the sandy substrate (*p* < 0.05) than pre-smolts and post-smolts (*p* < 0.05). Thus, in the dark environment, individual pre-smolts preferred the sandy substrate and stayed away from the small and large gravel substrate; individual post-smolts preferred the sandy substrate and stayed away from the large gravel substrate; and juveniles preferred the medium gravel substrate and stayed away from the sandy substrate and the small gravel substrate (Figure 10).

Substrate preferences of *B. tsinlingensis* individuals were similar in light and dark environments, with pre-smolts preferring sandy substrate, juveniles preferring medium gravel substrate, and post-smolts preferring not only sandy substrate but also medium gravel substrate in light environments.

#### 3.2.3. Results of Population Experiments in Light Environment

**Figure 11:**
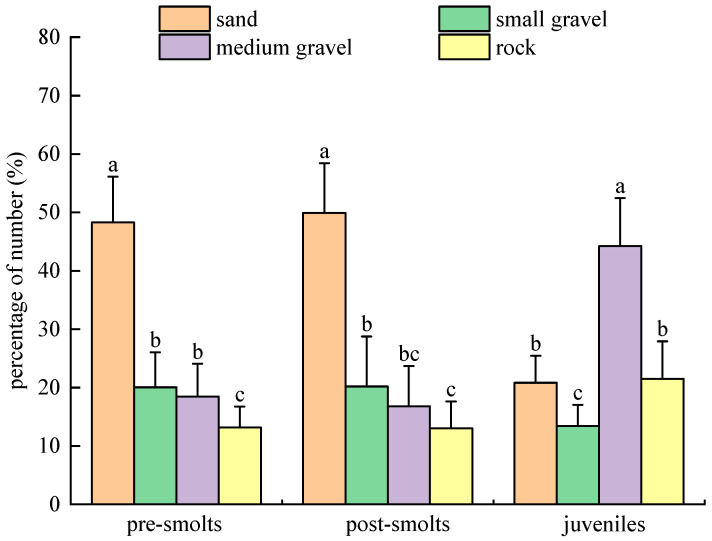
*B. tsinlingensis* substrate types (population experiment in light environment). (N = 160, different letters mean significant difference *p* < 0.05).

In population experiments with substrate types in the light environment (10–60 lx) for *B. tsinlingensis*, the percentages of the number of tails in the distribution that pre-smolts, post-smolts, and juveniles spent in the area of four substrate types (rock: 12–16 cm diameter; medium gravel: 5–7 cm diameter; small gravel: 1–2 cm diameter; and sand: <0.5 cm diameter) are as follows: the percentage of the distribution number of tails for pre-smolts: (13.17 ± 3.60)% for rock, (18.50 ± 5.57)% for medium gravel, (20.04 ± 6.01)% for small gravel, and (48.29 ± 7.83)% for sand; the percentage of the distribution number of tails for post-smolts: (13.04 ± 4.59)% for rock, (16.83 ± 6.86)% for medium gravel, (20.21 ± 8.56)% for small gravel, and (49.92 ± 8.51)% for sand; the percentage of the distribution number of tails for juveniles: (21.50 ± 6.41)% for rock, (44.21 ± 8.23)% for medium gravel, (13.46 ± 3.60)% for small gravel, and (20.83 ± 4.63)% for sand. Pre-smolts and post-smolts had the highest (*p* < 0.05) number of tails distributed in the sand substrate area, followed by small gravel substrate, medium gravel substrate, and the lowest number of tails distributed in rock substrate; juvenile fish had the highest (*p* < 0.05) number of tails distributed in the medium gravel substrate and the lowest (*p* < 0.05) number of tails distributed in the small gravel substrate area. With growth and development, the number of tails of juveniles in the middle gravel substrate increased sharply (*p* < 0.05) and was significantly more than that of pre-smolts and post-smolts; the number of tails of juveniles in the sandy substrate decreased significantly (*p* < 0.05). Therefore, in the light environment, the pre-smolt and post-smolt groups preferred sandy substrate and stayed away from rock substrate, and the juvenile individuals preferred medium gravel substrate and stayed away from small gravel substrate (Figure 11).

#### 3.2.4. Results of Population Experiments in Dark Environment

**Figure 12:**
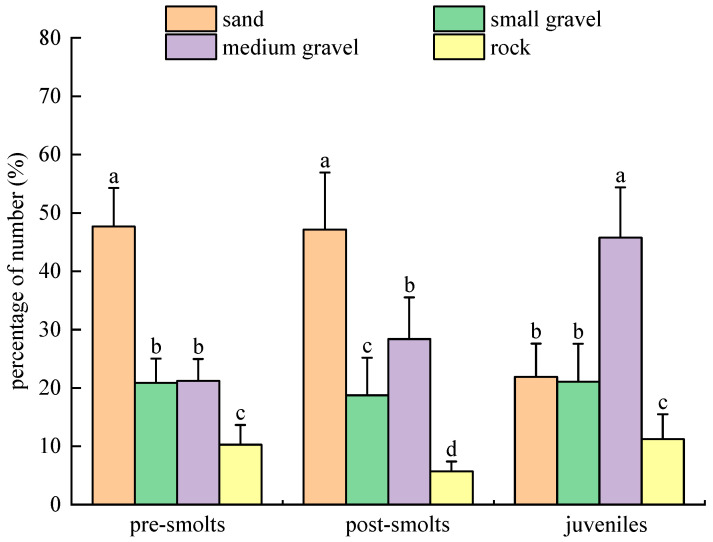
*B. tsinlingensis* substrate types (population experiment in dark environment). (N = 160, different letters mean significant difference *p* < 0.05).

In population experiments with substrate types in the dark for *B. tsinlingensis*, the percentages of the number of tails in the distribution that pre-smolts, post-smolts, and juveniles spent in the area of four substrate types (rock: 12–16 cm diameter; medium gravel: 5–7 cm diameter; small gravel: 1–2 cm diameter; and sand: <0.5 cm diameter) are as follows: the percentage of the distribution number of tails for pre-smolts: (10.29 ± 3.38)% for rock, (21.21 ± 3.75)% for medium gravel, (20.88 ± 4.15)% for small gravel, and (47.67 ± 6.59)% for sand; the percentage of the distribution number of tails for post-smolts: (5.71 ± 1.70)% for rock, (28.37 ± 7.16)% for medium gravel, (18.75 ± 6.43)% for small gravel, and (47.17 ± 9.77)% for sand; the percentage of the distribution number of tails for juveniles: (11.25 ± 4.25)% for rock, (45.75 ± 8.66)% for medium gravel, (21.08 ± 6.50)% for small gravel, and (21.92 ± 5.69)% for sand. Pre-smolts and post-smolts were similar, both with significantly higher (*p* < 0.05) numbers of tails distributed in areas of sandy substrate and significantly lower (*p* < 0.05) numbers of tails distributed in areas of rock substrate than other areas; juveniles had the highest number of tails in areas of medium gravel substrate (*p* < 0.05) and the lowest number of tails in large gravel (*p* < 0.05). Concerning growth and development, the number of tails of juvenile fish distributed in the mid-gravel substrate area increased dramatically and was significantly greater than that of pre-smolts and post-smolts (*p* < 0.05); the number of tails of juvenile fish distributed in the sandy substrate area decreased significantly (*p* < 0.05). Thus, in the dark environment, the pre-smolt and post-smolt populations preferred sandy substrate and avoided rock substrate, while the juvenile population preferred medium gravel substrate and avoided rock substrate (Figure 12).

In light and dark environments, *B. tsinlingensis* populations had the same substrate preferences, with smolts preferring sandy substrate and juveniles preferring medium gravel substrate.

## 4. Discussion

It is generally accepted that the behavior of organisms is the result of their long-term evolution and that the living environments and behavioral patterns chosen by fish reflect the results of their long-term evolutionary processes [25]. The preference of fish for substrate is actually a natural evolutionary manifestation of their choice of habitat [26]. Studies have shown that *Betta splendens* (Regan, 1910), *Poecilia reticulata* (Peters, 1859), and *Silurus asotus* (Linnaeus, 1758) significantly reduce their frequency of fighting on black substrate, decrease their stress response, and increase their feeding rate [27,28,29]. *Acipenser dabryanus* (Duméril, 1869), *Acipenser schrenckii* (Brandt, 1869), *A*. sinensis, and other sturgeons preferred white substrate because they have long inhabited shallow mudflats with abundant sunlight and high primary productivity, and the variety and quantity of bait organisms were abundant. White substrate is preferred by juveniles and early juveniles because it allows them to use their visual system to detect small baits that contrast with their environment and makes it easier for them to find and catch food [15,26,30]. The results showed that the pre-smolts, smolts, and juveniles of *B. tsinlingensis* showed a clear preference for black substrate under light and dark conditions and were observed to swim slowly and stay longer in aquariums with black substrate. This phenomenon is consistent with the preference of *Clarias gariepinus* (Burchell, 1822) [31], *Pseudobagrus fulvidraco* (Richardson, 1846) [32], and *A. japonic* [10], which rely on olfactory night feeding. Different substrate colors have different absorption capacities for light intensity, with black substrate absorbing significantly more light than white substrate and therefore reflecting a relatively lower light intensity. It has been shown that the choice of substrate color and phototaxis can occur simultaneously in fish. Early juvenile sturgeon tended to prefer white substrate, which also indicates that the preference for bright light areas coincides with their positive phototaxis [33], and the choice of black substrate by *A. japonica* indicates that the preference for low-light areas has a negative phototaxis [10]. Therefore, the preference for black substrate indirectly reflects the negative phototropism for light intensity, and the light intensity selection experiments in the present study demonstrated the negative phototropism of *B. tsinlingensis* in preferring low-light areas. The tendency of *B. tsinlingensis* to prefer black substrate and that of the *A. japonica* and early juvenile sturgeon to prefer a different substrate color are both reflective of habitat adaptations. Black substrate is preferred by *B. tsinlingensis*, possibly due to the fact that under light conditions, when light passes through the water and hits the black substrate, it creates a relatively dark water environment, which is conducive to providing a safe hiding environment. According to the feeding habits of *B. tsinlingensis*, it was found that the feeding effect was best at dusk or in darkness [19,21,34], but it was difficult to form a visual contrast with the bait in this environment, so it was not easy for *B. tsinlingensis* to rely on their vision to find food or catch bait, which indirectly indicated that the choice of a black substrate was more likely to be used as a way to find their own safe habitat to avoid predation and to hide themselves to facilitate surprise predation. It also suggests that black substrate may be chosen more as a safe habitat to avoid predation and to hide for surprise predation of live bait. *B. tsinlingensis* often choose deep-water areas in mountain streams with lush vegetation on both sides of the river [35], a high degree of shade, and a gravel substrate; when light passes through the habitat, it also creates a dim environment [35]. Therefore, the preference for black substrate is an adaptive choice of habitat, and its adaptive significance is mainly to avoid predation by enemies and to improve survival ability.

Juveniles are an important part of the early life cycle of fish. Juveniles at different stages of development have their own unique life patterns, nutritional composition, and growth characteristics, as well as different needs for different habitat conditions [36]. In the early growth stage of *P. fulvidraco*, fish between the ages of 1 and 18 days preferred to live in crevice environments, while fish between the ages of 19 and 25 days did not have this preference [37]. In this study, *B. tsinlingensis* smolts had a clear preference for sand and gravel substrate, and as they grew and developed, the juvenile stage had a clear preference for medium gravel substrate, showing that their smolts and juveniles showed differential selection of substrate type at different stages. The ability of fish in substrate selection is closely related to their developmental process [38]. In the early yolk sac stage (i.e., pre-smolts), the upper and lower jaws, digestive system, and sensory organs are not yet fully functioning, and the food intake during this period mainly focuses on absorbing nutrients from the yolk sacs; therefore, the food does not constitute a major factor limiting the choice of substrate type [39,40,41]. Nesting is a common behavior during the breeding season of fish, which requires the use of sandy substrate to form egg nests. Parents of *Oreochromis niloticus* (Linnaeus, 1758), *Gymnocypris przewalskii* (Kessler, 1876), and *Larimichthys crocea* (Richardson, 1846) build nests on sandy substrate to form egg nests. Pre-smolts are the first to come into contact with sandy substrate and retain their preference for them [42,43,44]. The parents of *B. tsinlingensis* often choose sandy substrate for “nest laying” during the breeding process. Pre-smolts hatch and develop in sandy substrate [35,45]. Therefore, the selection of sandy substrate by pre-smolts of *B. tsinlingensis* is not to search for abundant feed resources but to preserve the parents’ preference for sandy substrate. In post-smolts, the closure and opening of the upper and lower jaws of post-smolts are powerful, the digestive system begins to develop, the development of sensory organs is gradually perfected, feeding and absorbing exogenous nutrients are dominant, and the differentiation of swimming organs, such as fat and fins, is becoming increasingly obvious. At this time, juveniles already have the basic ability to swim [46], but late-stage juveniles of *B. tsinlingensis* do not have scales, and they choose the sandy substrate that is softer and flatter compared to the gravel substrate. Feeding in sand substrate can avoid fish body abrasion and help to maintain the integrity of smolt skin [20]. This is similar to the behavior of *Oncorhynchus keta* (Walbaum, 1792), *Salmo salar* (Linnaeus, 1758), and *Hucho bleekeri* (kimura, 1934), which also choose soft sandy substrate as their habitat during the fry stage [47,48,49]. The digestive system, sensory organs, and swimming organs of juvenile fish are well-developed, and their swimming ability is enhanced. They have the ability to choose according to their own preferences. The narrow crevice environment can provide a sense of security for fish, making it easy for them to quickly hide when enemies come. The juvenile stages of *Epinephelus akaara* (Temminck & Schlegel, 1842), *Procypris rabaudi* (Tchang, 1930), and *G. maculatum* have relatively weak self-defense capabilities and often inhabit narrow crevices between reef structures and gaps in gravel, exhibiting strong hiding behavior [17,50,51]. In the early field investigation of B. tsinlingensis, it was found that *B. tsinlingensis* also had strong hiding behavior, often hiding in the waterfall area where they lived in mountain streams [35]. Therefore, *B. tsinlingensis* juveniles chose medium gravel, and the crevices in the medium gravel provided good hiding space for juveniles, which is a natural evolutionary manifestation of habitat selection.

## 5. Conclusions

*B. tsinlingensis* is a cold-water fish with strong stress sensitivity. Based on research results and combined with the early biological characteristics of *B. tsinlingensis*, it is recommended to use black or dark substrate in the breeding process of *B. tsinlingensis* seedlings to provide a relatively safe, dark environment and avoid long-term stress. During the larval fish stage, a sandy substrate should be provided to protect their skin and facilitate rapid adaptation to the environment. During the juvenile fish stage, a medium gravel environment should be provided to offer suitable hiding places. It is important to note that due to the small, thin, and densely arranged scales of *B. tsinlingensis*, their skin has weak protective capabilities. Therefore, the substrate surface must be kept smooth to avoid skin abrasions and ulcers caused by frequent friction.

## Figures and Tables

**Table 1:** Length and weight of experimental fish at different stages (N = 30).

Fish	Length Range/cm	Length/cm	Weight Range/g	Weight/g
Pre-smolts	1.82~2.07	1.94 ± 0.12	0.04~0.07	0.05 ± 0.01
Post-smolts	2.12~2.20	2.17 ± 0.03	0.06~0.09	0.07± 0.01
Juveniles	3.54~3.62	3.59 ± 0.03	0.37~0.42	0.39 ± 0.02

## Data Availability

Data will be made available on request.
